# Glycine Promotes the Survival of a Subpopulation of Neural Stem Cells

**DOI:** 10.3389/fcell.2018.00068

**Published:** 2018-07-11

**Authors:** Abdelhamid Bekri, Pierre Drapeau

**Affiliations:** ^1^Research Center of the University of Montreal Hospital Center, University of Montreal, Montreal, QC, Canada; ^2^Department of Biochemistry, University of Montreal, Montreal, QC, Canada; ^3^Department of Neurosciences, University of Montreal, Montreal, QC, Canada

**Keywords:** zebrafish, glycine signaling, NSCs, nestin, survival, neurogenesis

## Abstract

Glycine is mainly known as an inhibitory neurotransmitter in adult mature neurons, regulating neuronal network activity in the central nervous system. In contrast, during embryogenesis glycine can act as an excitatory neurotransmitter and generates the first electrical signal in immature neurons. The roles and functional significance of this excitatory glycinergic activity during neurodevelopment are still unclear. Using the zebrafish embryo as a model, we previously showed that glycine regulates proliferation and differentiation of neural stem cells (NSCs) to interneurons. Moreover, we identified that glycine signaling in NSCs is associated with several common developmental pathways and surprisingly also the p53-related apoptosis. Here we investigated how glycine signaling regulates NSC survival. First, we showed by two approaches, acridine orange staining and active caspase 3 immunostaining that defects in glycine signaling induce an early and transient cell death, which was suppressed by knockdown of p53. Then, we developed an NSC transplantation strategy to directly assess NSC-autonomous development upon perturbing glycine signaling. *In vivo* time-lapse imaging showed that disruption of glycine signaling disturbed the normal NSC interkinetic nuclear migration, leading to cell cycle arrest and apoptosis. Finally, we analyzed two main subpopulations of NSCs, expressing either nestin or GFAP, by *in situ* labeling and in transgenic lines expressing GFP in either population. We found that disruption of glycine signaling induced a drastic and selective loss of nestin-positive (nestin^+^) NSCs, which was only partially rescued upon p53 knockdown. Taken together, our findings support a role of glycine signaling in promoting survival of the nestin^+^ NSC subpopulation early during development.

## Introduction

Glycine signaling plays a major role in the inhibition of mature neuronal circuits. In contrast, glycinergic activity in the embryo is excitatory and generates the first electrical signal in immature neurons and NSCs (Brustein et al., 2013; Mccracken et al., 2017). This switch in glycinergic activity is due to the late expression of the potassium chloride co-transporter 2, or KCC2 (Ben-Ari, 2002). In humans, disruption of the excitatory/inhibitory balance leads to the dysfunction of cognitive processes (Mohler, 2009) and presents a common feature among different developmental and neurological disorders such as autism, epilepsy, and hyperekplexia (Eichler et al., 2008; Bode and Lynch, 2014; Pilorge et al., 2016). However, many fundamental questions remain unanswered about the role of the switch in glycinergic activity in neurogenesis.

Neurogenesis is a dynamic process that is maintained by a precise balance of the cell cycle during division and differentiation of NSCs. This balance is regulated through an array of extrinsic and intrinsic molecular mechanisms (Schmidt et al., 2013). In an effort to investigate the roles of excitatory glycinergic activity during neurogenesis, we have used the zebrafish embryo as a model as it allows the disturbance of glycinergic activity by genetic and pharmacological approaches such as knockdown of the embryonic glycine receptor gene (GlyR) by anti-sense morpholino (MO) (Mcdearmid et al., 2006), blocking glycinergic activity with the GlyR antagonist strychnine (Cote and Drapeau, 2012), or overexpressing KCC2 from the onset of embryogenesis to reverse glycinergic activity (Brustein et al., 2013). We reported that disruption of glycine signaling by all of these approaches induces a drastic reduction (by half) in the number of interneurons, accompanied by an increase in the number of proliferating cells as assayed by phospho-Histone 3 (pH3) immunostaining (Cote and Drapeau, 2012). We also showed that glycinergic activity regulates spontaneous calcium transients in Glial Fibrillary Acidic Protein positive (GFAP^+^) NSCs during embryonic development (Brustein et al., 2013). We therefore hypothesized that glycinergic activity plays an important role in neurogenesis by modulating NSC development.

In order to understand the molecular basis of NSC regulation by glycine signaling, we previously performed a transcriptomic analysis of the GFAP^+^ NSC population upon disrupting glycine signaling and our results highlighted five main pathways modulated by glycine signaling in NSCs, those for calcium, BMP, SHH, TGF-beta and the p53 pathway (Samarut et al., 2016). While the first four signaling pathways are well established in embryogenesis, modulation of the p53 pathway mediating apoptosis was to us a surprising result as little cell death normal occurs during zebrafish embryogenesis. To investigate the potential role of glycine signaling in NSC survival during development, we assayed apoptotic cells and found that disruption of glycinergic activity induced an early, transient loss of NSCs. Second, to directly assess at a cellular level the role of glycine signaling during neural development, we tracked NSC-autonomous glycinergic activity and showed that its disruption induced the loss specifically of the nestin^+^ NSC subpopulation. In sum, we report that glycine signaling plays a crucial role during neuronal development by promoting the survival of the nestin^+^ subpopulation of NSCs.

## Materials and methods

### Zebrafish

Zebrafish (Danio rerio) were maintained at 28°C under a 12-h light/dark cycle in the CRCHUM Zebrafish Facility and they were raised and manipulated as per guidelines of the Canadian Council for Animal Care (Westerfield, 2007). Embryos were staged as described previously (Kimmel et al., 1995). The following genetic strains were used: *tg(-3.9nestin:GFP)* and *tg(GFAP:GFP)* (provided by Jana Maier—Karlsruhe Institute of Technology, Germany), *tg(HuC:Gal4)* (provided by Higashijima, Shin-ichi—Okazaki Institute for Integrative Bioscience, Japan).

### Microinjection

Embryos at one cell stage were injected with MO (Gene tools), to knockdown a zebrafish embryo GlyR a4a subunit by Glr-MO, knockdown of p53 by p53-MO (Gene tools) and mismatched MO used as control (Ctrl-MO). Sequences of MOs were designed as previously described (Mcdearmid et al., 2006; Cote and Drapeau, 2012; Samarut et al., 2016).

### Apoptosis assay and cell death quantification

To assay cell death, *in vivo* AO staining was performed in live embryos at 14, 20, 24, and 48 h post-fertilization (hpf). The stock solution of AO (5 mg/ml, 300x) was diluted in egg water to 1X concentration. Then, embryos were dechorionated and incubated with AO (1X) in the dark for 10 min. The reaction was then stopped by washing the embryos several times with eggs water and the embryos were imaged using a spinning-disk confocal microscope. Acquisition of images was performed with Volocity software (Perkin–Elmer). Cell death was quantified by counting AO-positives (AO^+^) cells in the first five somites of each zebrafish spinal cord. Statistics test was performed by one-way ANOVA analysis by compared the changing of the AO^+^ cells numbers in the different conditions. In addition, cell death was confirmed by labeling aCas3. Embryos injected by Ctrl-MO or Glr-MO was fixed overnight at 20 or 24 hpf by 4% formaldehyde (4% PFA) at 4°C, then whole-mount immunohistochemistry was conducted as previously described (Sorrells et al., 2013). Primary antibodies rabbit anti-active Caspase-3 (Fisher Scientific, Cat. # BDB559565) was used at (1:500 dilution). Then, secondary Alexa Fluor 488 anti-Rabbit IgG (Life Technologies, Cat. # A10042) was used at (1:2,000 dilution).

### Whole-mount *in situ* hybridization

Embryos injected with Glr-MO or Ctrl-MO were dechorionated and washed twice with phosphate-buffered saline (PBS) for 5 min each at room temperature and then fixed immediately overnight in cold 4% PFA at the desired stage. Embryos were then dehydrated in a gradient of methanol (25, 50, and 75%, methanol in PBS) and transferred into 100% methanol and stored at −20°C. The embryos were then hydrated by inverted gradients and loaded into sample holders and mounted into an *in situ* hybridization device (Folgentec-France). Embryos were subjected for 22 h to a continuous stream (1.7 ml/min) of successive reagents following the *in situ* hybridization protocol as previously described (Bekri et al., 2014). Finally, enzymatic detection was performed in PM purple reagent (Roche) under constant observation.

### Probes, cell labeling, and transplantation

**Figure 1:**
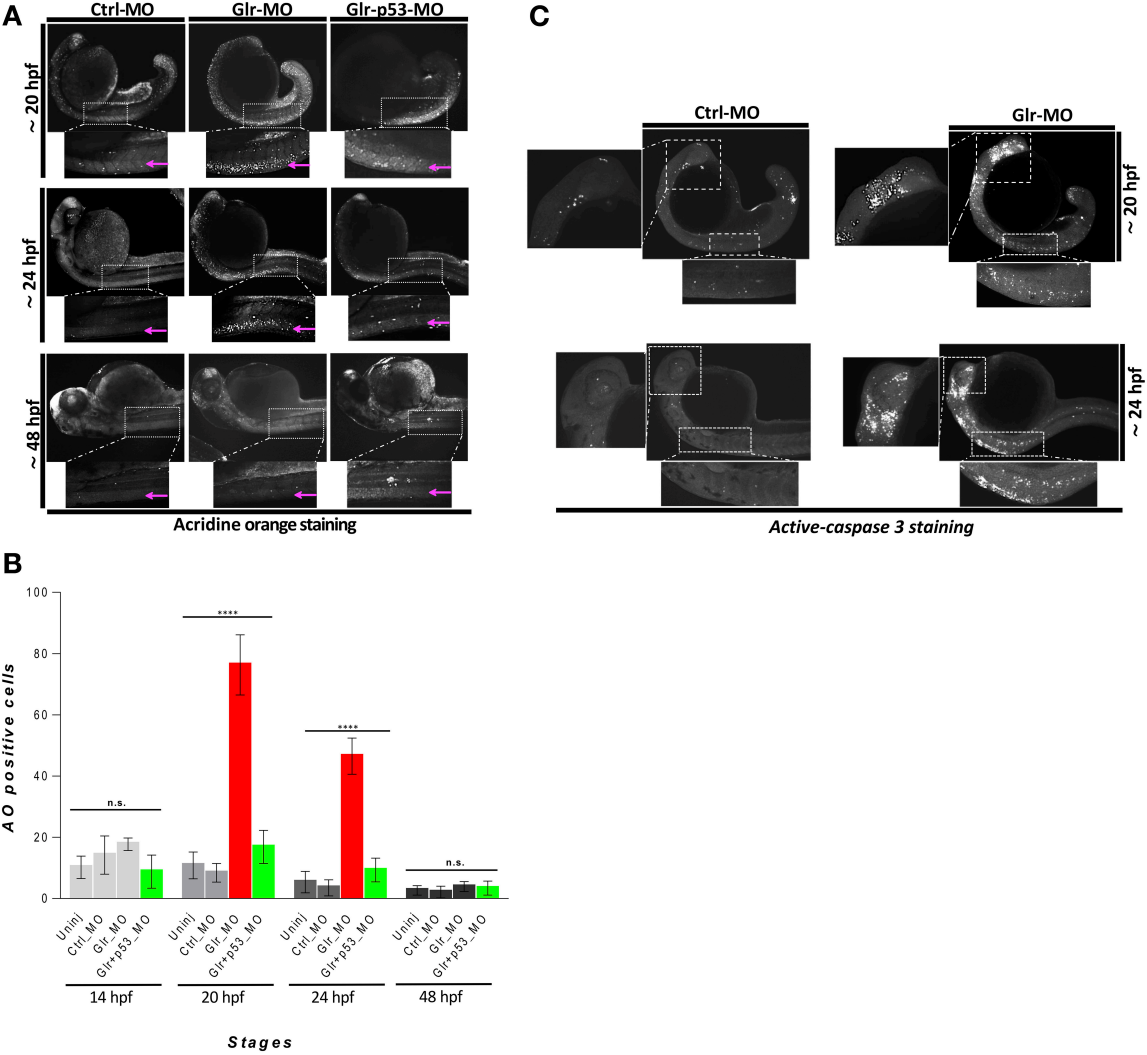
Disruption of glycine signaling causes an early and transient neural cell death during embryonic development. **(A)** Lateral views of zebrafish embryos stained by AO. live embryos injected by Ctrl-MO, Glr-MO, and p53-Glr-MO were stained by AO and imaged at 20, 24, and 48 hpf, higher magnification of cell death at spinal cord regions were highlighted in dotted boxed area. **(B)** Lateral view of zebrafish embryos at 20 and 24 hpf embryos injected by Glr-MO or Ctlr-MO and labeled by immunostaining for aCas3 (white dots), dotted box in the left and bottom showed a magnification of positives aCase3 in the brain and spinal cord. **(C)** Cell death quantification during zebrafish embryos development. AO positive cells at the spinal cord were quantified and compared in each condition, uninjected, Ctrl-MO, Glr-MO and p53-Glr-MO injected embryos at four-time points, 14, 20, 24, and 48 hpf. One-way ANOVA statistical analysis was performed (*n* = 15, ^****^p < 0.0001).

To make probes for *GFAP* and *nestin*, total RNA was isolated from 24 hpf zebrafish embryos. Then, total mRNA was reverse transcribed to cDNA. A specific primers were designed to target around 1000 bp of nestin or GFAP RNAs according to previous descriptions (Thisse and Thisse, 2008). The sequences of primers used in synthesizing probes are as follows: nestin forward (F):5′-CCATGCAGCAAAGAGAAGAA-3′, nestin revers (Sp6-R): 5′-TAATACGACTCACTATAGGG-TGTGACTTGTAGACACAGAACTGC-3′, GFAP (F): 5′-GAGATCAATTTCCTGAAGAAGGTC-3′ and GFAP (Sp6-R): 5′-TAATACGACTCACTATAGGGCTGCACCGGAACAGTGATT-3′. To labeling nuclei and cell membranes, sense strand-capped RNAs were synthesized by using SP6 transcription mMESSAGE mMACHINE system (Ambion) from NotI-linearized plasmids including, pCS2-H2B-GFP, pCS2-H2B-RFP that expressed Histone 2B fused with FGP or RFP proteins which used as nucleic markers, or pCS2-CAAX-GFP that expressed farnesylated GFP which used as Membrane markers (kind gift from Jonathan DW Clarke, King's College London, UK) (Alexandre et al., 2010). Then, nuclei and cell membranes were labeled respectively by co-injection embryos with 150 ng of mRNAs encoding either fluorescent H2B fusion protein (H2B-RFP or H2B-GFP) and membrane marker (CAAX-GFP). Transplantation was performed as previously described (Kemp et al., 2009). Donor embryos were co-injected at one-cell stage with the nuclear and membrane markers and Glr-MO or Ctrl-MO, following strategy in **Figure 3Ai**. At the shield stage (6 hpf), 10 to 20 cells from each donor embryo were transplanted into the same wild type (wt) host embryo (Figure 1A).

### Confocal and time-lapse imaging

Host embryos were anesthetized with 0.03% tricaine (MS222; Sigma) at 18 hpf and mounted in a small dish with 1.5% low-melting agarose (Sigma); the embryos were dorsally oriented so that marked cells located in the posterior hindbrain and spinal cord were facing up. Then, the dish with embryos was maintained at 28°C using a heating plate system (Live Cell Instrument). Images were acquired using a Quorum Technologies spinning-disk confocal microscope with a 40X water-immersion objective. Time-lapse imaging was performed by taking a series of z stacks every 5 min during 10 h. Data sets were analyzed with Volocity® software.

## Results

### Early, transient NSC survival defects upon disruption of glycine signaling

As the zebrafish interneuron population is broadly reduced upon disruption of glycinergic activity (Mcdearmid et al., 2006; Cote and Drapeau, 2012), we hypothesized that it plays an early role during neurogenesis by regulating a common progenitor. Recently, we sorted by flow cytometry the GFAP^+^ NSCs from zebrafish embryos and analyzed these by RNA-sequencing, which revealed that glycine regulates five main pathways in NSCs, including surprisingly the p53 pathways (Samarut et al., 2016). As cell death was not observed upon blocking glycinergic activity by a selective morpholino (Glr-MO) in zebrafish embryos when examined at a late stage of 48 hpf (Mcdearmid et al., 2006), more than a day following the onset of neuronal differentiation and just prior to hatching, we reasoned that glycine might affect survival of earlier NSCs.

To investigate this hypothesis, we first performed an *in vivo* time-course analysis of cell death by acridine orange (AO) staining. Surprisingly, we found that embryos injected with Glr-MO had a large number of AO^+^ cells compared with embryos injected with control MO (Ctrl-MO) and these AO^+^ cells were specifically located in the CNS (Figure 1A). To confirm these results, we performed another apoptosis essay based on detection by immunostaining of activated Caspase 3 (aCas3), which is known as a major mediator of programmed cell death (Taylor et al., 2008). As with the AO staining, we detected many aCas3 positive cells in the brain and spinal cord only upon disruption of glycine signaling (Figure 1B). Importantly, quantification of the number of AO^+^ cells over four different time-points (14, 20, 24, and 48 hpf) showed a transient cell loss between 20 and 24 hpf compared with control embryos (approximately 4-folds). However, no significant cell death was noted at 14 and 48 hpf (Figure 1C). To verify whether cell death is related to overexpression of p53 as reported by Samarut et al. (2016), we down regulated p53 expression upon disruption glycine signaling by co-injecting p53-MO and Glr-MO in the same embryos, followed by a time-course analysis of cell death by AO staining. The results showed a significant reduction of cell death observed between 20 and 24 hpf upon disrupting glycine signaling without any difference in cell death at 14 and 48 hpf (Figure 1C).

These results suggest that p53 signaling is related to cell death induced by glycine signaling disruption. These findings are in agreement with (Mcdearmid et al., 2006) with regards to the absence of the cell death during late stages of zebrafish development (48 hpf) and related the p53 gene up-regulation observed by Samarut et al. (2016) at 20 hpf. Additionally, we reveal that glycine signaling disruption induces a transient cell death between 20 and 24 hpf at CNS. Taken together, these experiments demonstrated that disruption of glycine signaling induce a transient programmed cell death in the CNS which is related to overexpression of p53.

### Cell-autonomous NSC phenotype upon disruption of glycine signaling

Although our results show that defective glycine signaling induced cell death in the CNS, they do not demonstrate which kinds of neural cells are affected. To better clarify the role of glycine signaling at a cellular level during neural development, we developed an approach based on single-cell transplantation coupled with *in vivo* time-lapse confocal imaging. This approach allowed us (1) to determine the cell-autonomous activity of glycine signaling (2) define when and which kinds of neural cells are affected, and (3) compare control and knockdown cells in the same environment. To do so, we used as a donor embryo the *tg(HuC:Gal4,UAS:RFP)* line in which the pan-neuronal HuC/Elav3 promoter drives RFP expression (Kim et al., 1996). During the time lapse, mature neuronal fate was assigned if these cells expressed RFP in the cytoplasm and nuclei, or NSC fate if they did not express RFP, with an example shown in (Supplementary Figures 1A,B).

**Figure 2:**
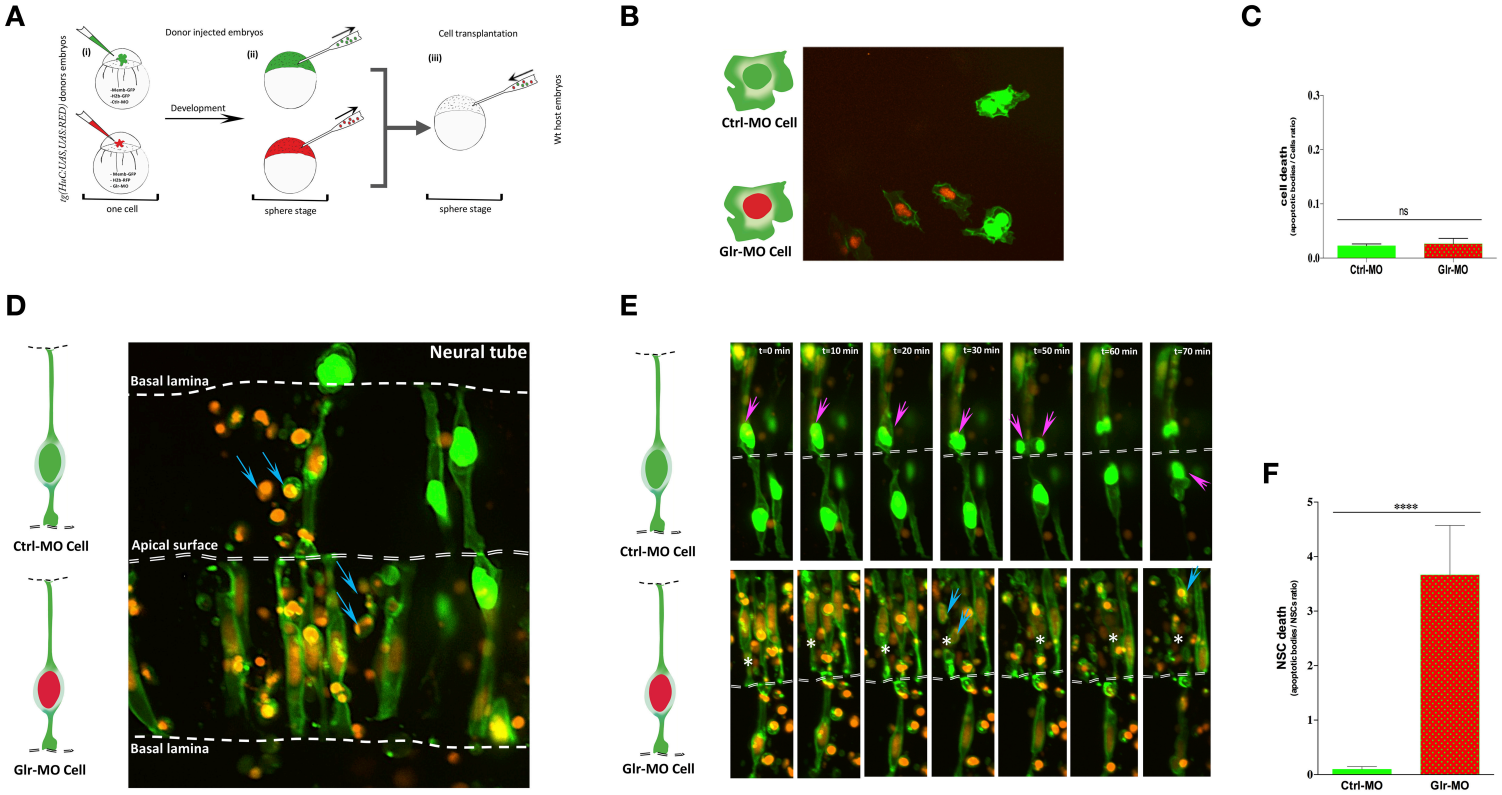
Defects of cell-autonomous glycine signaling induce a specific NSCs loss. **(A)** Cell transplantation strategy. Donor *tg(HuC:Gal4,UAS:RFP)* was injected at one-cell by mRNA to target membrane, cell nucleus and Glr-MO or Ctrl-MO as indicated in step (i). At the sphere stage, cells from both conditions were transplanted together in the same host wt embryo in the blastoderm margin (ii and iii). **(B)** Confocal z-series images of transplanted NSCs. Ctrl-MO transplanted cells showed a normal development to NSCs (NSCs with green membranes and nuclei). However, a considerable of apoptotic bodies were observed in Glr-MO transplanted cells (NSCs with green membranes and red nuclei). The basal and apical surface is outlined by a dotted line and double lines respectively. **(C)** Live imaging revealed programmed death of NSCs upon glycine signal disruption. Selected frames from 2 h time-lapse sequence of transplanted NSCs development showed a normal interkinetic nuclear migration of Ctrl-MO NSCs followed by cell division (top sequence, magenta arrows). In contrast, some of Glr-MO NSCs showed an arrest movement of interkinetic nuclear migration (bottom sequence, asterisk), followed by fragmentation of NSC to apoptotic bodies (bottom sequence, blue arrows). **(D)** Quantification of transplanted NSCs death (apoptotic bodies / NSC ratio) in both conditions, Glr-MO (and Ctrl-MO reveal a drastic NSCs death upon disruption of glycine signaling by more than folds. **(E)** Disruption of glycine signaling does not affect non-neuronal cells survival. Transplanted Glr-MO cells (red nuclei) and Ctrl-MO cells (Green nuclei) were developed naturally to non-neural cells (skin) without showing apoptotic bodies formation. **(F)** Quantification of transplanted non-neural cell in both conditions, Glr-MO and Ctrl-MO reveal no significant cell death upon disruption of glycine signaling. *t*-test statistical analysis was performed (*n* = 6, ^****^p < 0.0001).

As a first step, donor eggs from *tg(HuC:Gal4; UAS:RFP)* embryos were triply injected with Ctrl or Glr-MO, mRNA coding for the membrane marker (CAAX-GFP) and a nuclear marker (H2B-GFP or RFP) following to the strategy shown in Figure 2Ai. Accordingly, **control** donor embryos were injected with Ctrl-MO, mRNA coding for H2B-GFP and CAAX-GFP (**Ctrl-MO****, GFP**^+^, **RFP****^−^)**, whereas **knockdown** donor embryos were injected with Glr-MO, mRNA coding for H2B-RFP and CAAX-GFP **(****Glr-MO****, GFP**^+^,**RFP**^+^**)**. Thus, control cells would have green nuclei whereas GlyR knockdown cells would have red nuclei and both would be delineated by green membranes. Second, at desired stages and according to the fate map described previously (Kimmel et al., 1990), donor cells from control and knockdown embryos were transplanted together into an area of host wt embryo fated to develop into the CNS (Figure 2Aii,iii). Finally, at 18 hpf neuron development in wt hosts was recorded by confocal time-lapse imaging.

At 18 hpf, the host embryos showed that transplanted cells naturally migrated into the neural tube and developed into neuroepithelial/radial glial NSCs (Figure 2D). Time-point stacked images showed a large number of apoptotic bodies containing condensed DNA from cells with red nuclei, indicating that transplanted knockdown cells **(****Glr-MO****, GFP**^+^, **RFP**^+^**)** were dying (Figure 2D, blue arrows) compared to with control transplanted cells **(****Ctrl-MO****, GFP**^+^, **RFP**^−^**)**, which continued to divide and gave rise to new daughter cells (Figure 2E, top sequence, magenta arrows). Quantification of NSC death revealed a dramatic increase of NSC death upon disruption of glycine signaling compared with control condition (more than 10-folds) (Figure 2F). Closer examination revealed that transplanted NSCs showed a normal internuclear kinetic movement (INM) in control NSCs **(****Ctrl-MO****, GFP**^+^, **RFP**^−^**)** followed by cell division (Figure 2E, top sequence, magenta arrows; Gotz and Huttner, 2005), whereas some of the GlyR knockdown NSCs **(****Glr-MO****, GFP**^+^, **RFP**^+^**)** showed an arrested INM during mitotic G2-M phases (Figure 2E, bottom sequence, asterisk) followed by cell fragmentation to apoptotic bodies (Figure 2E, bottom sequence, blue arrows). To confirm the specific effect of glycine signaling to NSCs and rule-out a general toxicity of MO injections, both knockdown **(****Glr-MO****, GFP**^+^, **RFP**^+^**)** and control **(****Ctrl-MO****, GFP**^+^, **RFP**^−^**)** cells were transplanted into the area of the host wt embryo that gives rise to non-neural cells such as skin or yolk cells. The results showed a normal fate development of both transplanted cells as they gave rise to skin cells in the tail and yolk region (Figure 2B). Quantification of non-neural cell death showed no significant cell death in both conditions (Figure 2C). Thus the apoptotic effect of the GlyR-MO was selective for NSCs.

To verify that NSC death was independent of mRNA nuclear labeling, we switched the injection of H2B-GFP during knockdowns and H2B-RFP during control while keeping the injection of CAAX-GFP mRNA as membrane makers in both conditions. The results revealed the same phenotype, with a large number of apoptotic bodies containing green nuclei which present in this case the GlyR knockdown condition compared with control condition which showed a normal development of NSCs with red nuclei (Supplementary Figure 1C). As an additional control, we transplanted this GlyR knockdown NSCs with green nuclei in non-neuronal areas and observed normal development of cells in the skin without fragmentation to apoptotic bodies (Supplementary Figure 1D). These results showed the independence of cell death from mRNA nuclear labeling and confirmed the specificity of NSC death upon glycine signaling disruption. Taken together, these findings demonstrate that glycine signaling plays a specific and major role in NSC survival as reduction of this activity leads to the loss kind of NSCs.

### The nestin^+^ NSC subpopulation is lost upon disruption of glycine signaling

To define which kind of NSC is lost when glycinergic activity is blocked, we analyzed the patterns of the expression of Glial Fibrillary Acidic Protein (GFAP) and neuroectodermal stem cell protein (nestin) as two main markers of NSC subpopulations (Wiese et al., 2004; Kim et al., 2011). In addition to being NSC markers, these proteins belong to the intermediate filament protein family, which plays a major role in the organization of cell structure and dynamics (Sanghvi-Shah and Weber, 2017). To examine these subpopulations, we used two different approaches: (1) whole-mount *in situ* hybridization and (2) double transgenic lines that express GFP and RFP in different cells.

**Figure 3:**
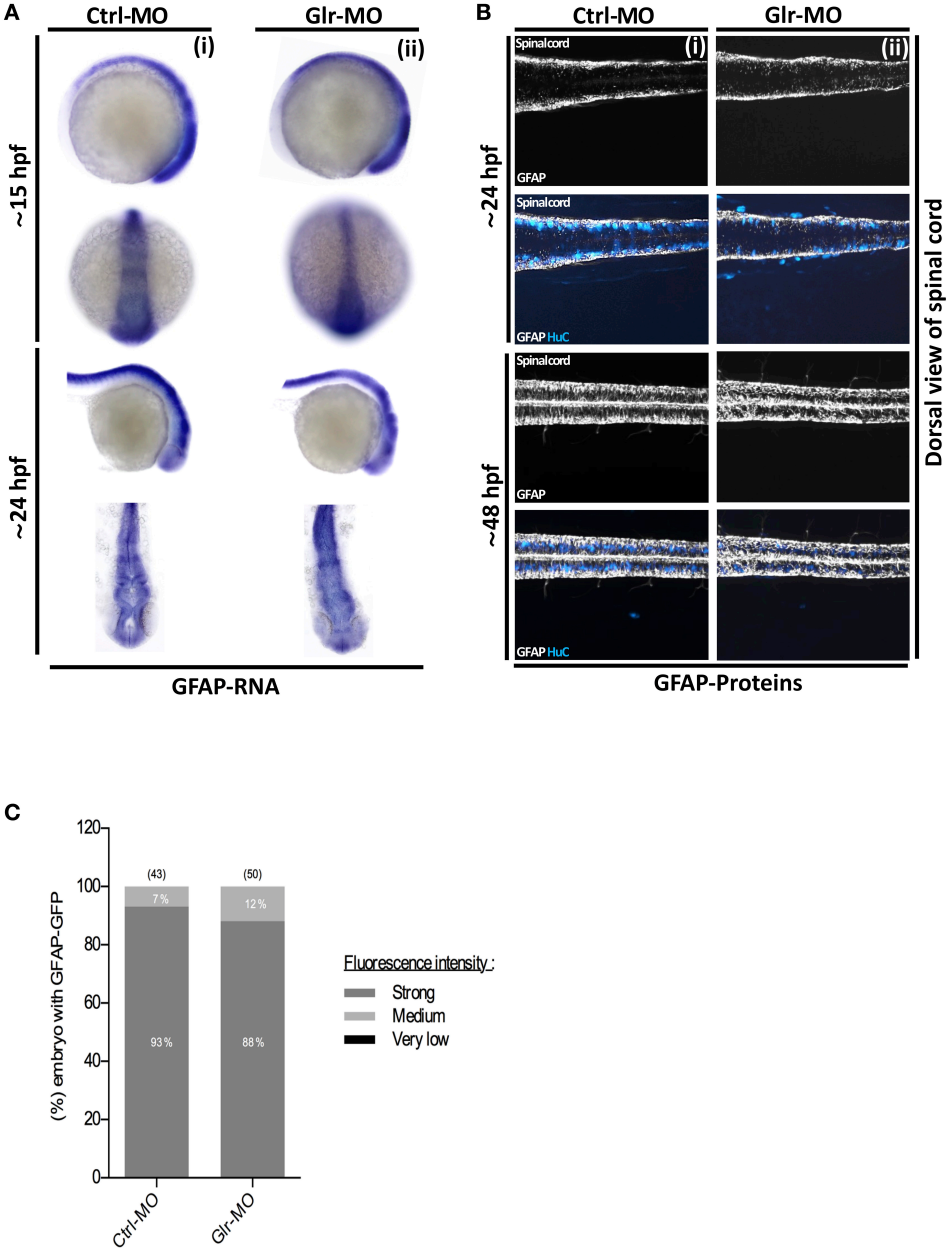
Glycine signaling defect does not affect GFAP^+^ NSCs subpopulation. **(A)** Using GFAP as a marker of NSCs subpopulation, GFAP transcripts were analyzed by whole-mount *in situ* hybridization. No major difference of GFAP^+^ subpopulation was observed between Ctrl-MO (i), and Glr-MO (ii) conditions at 15 and 24 hpf. **(B)** Double *tg(GFAP:GFP; HuC:RFP)* line was used to analysis GFAP expression (white) and HuC^+^ expression as mature neurons marker (Bleu). GFAP^+^ signal does not change between Ctrl-MO (i) and Glr-MO (ii) conditions at 24 and 48 hpf. However, a clear decreasing of HuC^+^ signal was observed between Ctrl-MO (i), and Glr-MO (ii) conditions. **(C)** Quantification of GFAP-GFP embryos upon disruption of glycine signaling does not affect GFAP^+^ subpopulation. For both ISH and IHC, *n* > 20 embryos per sample. IHC, immunohistochemistry; ISH, in situ hybridization.

**Figure 4:**
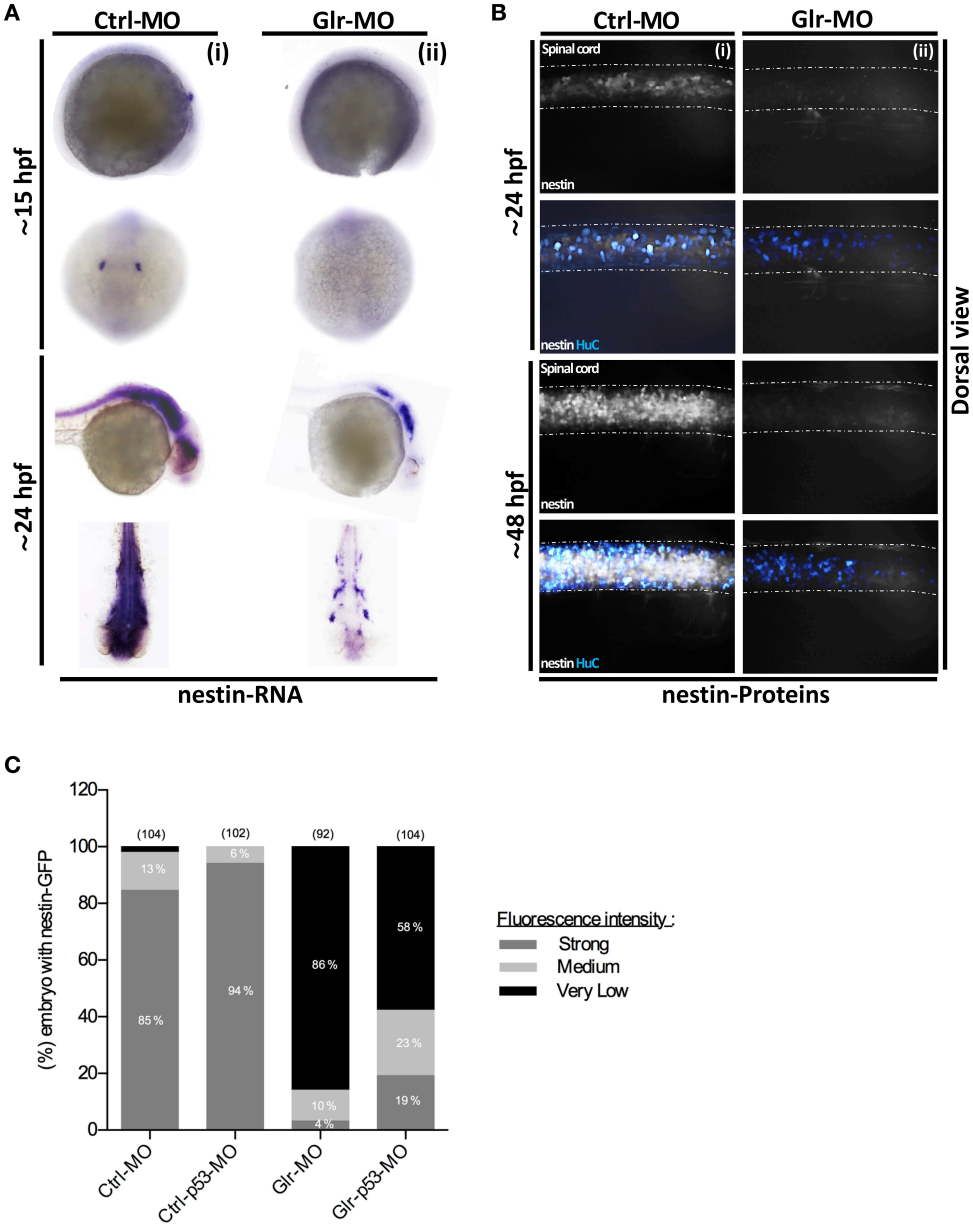
Glycine signaling defects induces a drastic loss of nestin^+^ NSCs subpopulation. **(A)** Using nestin as a marker of NSCs subpopulation, nestin transcripts were analyzed by whole-mount *in situ* hybridization. A drastic loss of nestin^+^ subpopulation was observed between Ctrl-MO (i), and Glr-MO (ii) conditions at 15 and 24 hpf. **(B)** Double *tg(GFAP:GFP; HuC:RFP)* line was used to analysis nestin (white) and HuC^+^ (Bleu) expressions. Major reduction of nestin^+^ signals in Glr-MO conditions (ii) compared with Ctrl-MO (i) at 24 and 48 hpf. In addition to reduction of HuC+ signals in Glr-MO condition (ii). **(C)** Quantification of nestin-GFP embryos upon disruption of glycine signaling reveal a drastic reduction of GFP signal compared with control. However, co-injecting of p53-MO and Glr-MO rescue partially nestin^+^ subpopulation. For both ISH and IHC, *n* > 20 embryos per sample. IHC, immunohistochemistry; ISH, in situ hybridization.

To perform *in situ* labeling, specific anti-sense RNA probes targeting GFAP or nestin zebrafish mRNAs were designed and synthesized. Then, control and knockdown embryos at 15 and 24 hpf were fixed and processed for *in situ* hybridization. GFAP *in situ* labeling showed a broad GFAP-positive (GFAP^+^) sup-population in the neural tube during early stages of embryonic development (15 hpf) and we noted that they maintained their distribution in the CNS as they were located broadly in the spinal cord and brain at 24 hpf (Figure 3Ai). As well, GFAP^+^ *in situ* labeling showed no difference between Ctrl and Glr-MO treated embryos at both 15 and 24 hpf stages (Figure 3Aii). In contrast, *in situ* labeling with the nestin transcript demonstrated a different pattern than that for GFAP^+^ labeling as nestin^+^ cells were detected only in the otic vesicle (OV) and were greatly reduced in the neural tube at 15 hpf. At 24 hpf, they were widely present in the brain and spinal cord (Figure 4Ai) of Ctrl-MO embryos. Importantly, Glr-MO treated embryos showed a drastic reduction of the nestin^+^ subpopulation at in the OV and CNS at 15 and 24 hpf, compared with Ctrl-MO treated embryos which the nestin^+^ subpopulation was unaffected (Figure 4Aii).

To confirm our results, we examined the nestin and GFAP subpopulations in two double transgenic lines, *tg(GFAP:GFP;HuC:RFP)* and *tg(nestin:GFP;HuC:RFP)*. The GFP signal was used to follow the GFAP^+^or nestin^+^ subpopulations and the RFP signal was used to follow the mature neuron population upon disruption of glycine signal. In accordance with the *in situ* labeling results, GFAP-GFP cells in the *tg(GFAP:GFP;HuC:RFP)* embryos were broadly present in the CNS at 24 and 48 hpf (Figure 3B) without significant difference between percentage of GFAP-GFP embryos treated by Ctrl or Glr-MO (Ctrl-MO: 93%, *n* = 43; Glr-MO: 88%, *n* = 50) (Figure 3C). However, the analysis of nestin-GFP cells using the *tg(3.9nestin:GFP;HuC:RFP)* embryos showed a drastic reduction at 24 and 48 hpf of percentage of nestin-GFP embryos treated by Glr-MO compared with Ctrl-MO (Ctrl-MO: 85%, *n* = 104; Glr-MO: 4%, *n* = 92; Figures 4B,C), indicating a selective loss of the nestin^+^ subpopulation upon disruption of glycine signaling. Furthermore, the analysis of RFP signal indicating a loss of mature neurons which confirming the previous reports (Mcdearmid et al., 2006) (Figures 3Bii, **4B**ii).

To verify whether down regulation of p53 overexpressed upon disruption of glycine signaling by p53-MO could rescue the nestin^+^ subpopulation loss (Figures 1A,B), we co-injected p53-MO with Ctrl-MO or Glr-MO in the *tg(nestin:GFP;HuC:RFP)* and we analyzed nestin-GFP embryo. The results revealed no significant difference of nestin-GFP NSCs between Ctrl-MO and Ctrl-p53-MO (Ctrl-MO: 2%, *n* = 104; Ctrl-p53-MO: 0%, *n* = 102; Figure 4C). Nonetheless, a partial rescuing by about 28% of nestin-GFP embryos was observed by co-injecting Glr-p53-MO compared with Glr-MO (Glr-MO: 86%, *n* = 92; p53-Glr-MO: 58%, *n* = 104). These suggest that p53 signaling is related to glycine signaling mechanism to maintain nestin^+^subpopulation survival. However, down regulation of p53 alone did not fully rescue the nestin^+^ subpopulation upon disruption of glycine signaling, which might be explained by the major effect that glycine signaling disruption caused on the other pathways as reported by Samarut et al. (2016), and which might contribute together to maintain nestin^+^ subpopulation survival via glycine signaling. Taken together, these finding demonstrate that glycine signaling play a role in NSC development by promoting survival of the nestin^+^ subpopulation.

## Discussion

The neurotransmitter signaling has primarily been associated with the physiologic function of neurons. However, several reports have identified roles for neurotransmitters and their receptors in cell fate determination by modulating NSC proliferation or differentiation, including by γ-aminobutyric acid (GABA), glutamate and dopamine neurotransmitters (Baker et al., 2004; Yoshimizu and Chaki, 2004; Anjard and Loomis, 2006; Andang et al., 2008). In this work, we investigated the role of glycine as how its action as an excitatory neurotransmitter and the functional significance of its switch in activity during embryonic neurodevelopment is still limited. We explored by cellular approaches the transcriptomic data reported previously on pathways modulated by glycine signaling in NSCs (Samarut et al., 2016). We reveled that disruption of glycine signaling induced an early and transient NSC death related to p53 signaling. Further, we demonstrated by cell transplantation that NSC autonomous glycine signal disruption led to defects in cell division and caused cell death. In addition, we revealed that unlike the GFAP^+^ NSC subpopulation, glycine signaling was specially required for survival of the nestin^+^ NSC subpopulation.

### Glycine signaling suppresses programmed cell death in the CNS

We based our study on previous reports that disruption of glycinergic activity in early (20 hpf) embryonic development leads to overexpression of the apoptotic P53 gene (Samarut et al., 2016). This result was surprising as McDearmid et al. reported that no apoptotic neural death occurred at late (48 hpf) embryonic stages when glycine signaling was defective (Mcdearmid et al., 2006). To clarify the contradiction between these reports, we performed two different assays of cell death, AO staining and aCas3 immunostaining, at several developmental time points. Both approaches confirmed that glycine signaling controls the survival of NSCs at an early stage of development (20 hpf) as reported by Samarut et al. (Figures 1A,C). However, the time course of cell death as assayed by AO staining revealed that, unlike Ctrl-MO treated embryos which had few apoptotic cells, Glr-MO-treated embryos showed a transient time-window of cell death between 20 and 24 hpf of development and with no significant increase in cell death before (at 14 hpf) or later (48 hpf), as reported previously (Mcdearmid et al., 2006). Additionally, down regulation of p53, which is overexpressed upon disruption of glycine signaling, partially reduced the cell death observed between 20 and 24 hpf. These results suggest that glycine signaling is required for NSC survival during a precise time-window of development and p53 signaling is related to cell death induced by glycine signaling disruption.

Several functional studies implicate GlyRs in developmental processes in the CNS and more broadly in other organs. In the immature retina, glycinergic activity influences the development of rod photoreceptors (Young and Cepko, 2004). Also, glycine signaling was found to control the proliferation of progenitor cells during corticogenesis (Mcdearmid et al., 2006; Cote and Drapeau, 2012; Avila et al., 2014) and to promote the migration of cortical interneurons (Avila et al., 2013). However, its role in NSC survival is less well characterized. Several studies indicated that the chloride transporter KCC2 contributes to neuronal survival during and after toxic stress (Pellegrino et al., 2011; Allain et al., 2016). Likewise, in other organs glycine was reported reducing hepatic damage and improve the survival rate of endotoxemia mouse models by regulating TNF-a and IL-10 secretion (Bruck et al., 2003). Moreover, it was reported that in liver injury, glycine prevents apoptosis of sinusoidal endothelial cells under vascular endothelial growth factor (VEGF) deprivation (Zhang et al., 2000). Furthermore, administration of glycine improves survival in rat liver transplantation (Schemmer et al., 1999) and prevents apoptosis during mesenteric ischemia/perfusion injury by down-regulation of the death-inducing signals in rat models (Zhong et al., 2012). Thus, glycine appears to play ubiquitous roles in cell survival and differentiation.

To investigate glycine signaling, we previously validated three different approaches that yielded the same phenotype: KCC2 overexpression, strychnine agonists or anti-sense MO knockdown (Mcdearmid et al., 2006; Cote and Drapeau, 2012; Zhong et al., 2012; Brustein et al., 2013; Samarut et al., 2016), and in this study we selected anti-sense MO knockdown for its reproducible and robust phenotype. To minimize potential off-target effects, we performed control experiments such as dose-response and use of mismatched anti-sense MO as a ctrl-MO. The ctrl-MO and Glr-MO were used at a very low level (0.6 ng) and they did not cause a major morphological abnormality but rather a subtle but significant loss of NSC subpopulation. Then, we developed a cell transplantation strategy to determine the NSC autonomy of glycine signaling. Both ctrl-MO and Glr-MO treated NSCs were transplanted together in the same host wt embryo to evaluate cell behavior in the same (non-autonomous) environment. Time-lapse recording showed a normal behavior of ctrl-MO treated NSCs, which divided by INM to yield two daughter progenitor cells and in some cases neurons (Alexandre et al., 2010; Figure 2). In contrast many Glr-MO cells arrested during mitotic division and entered apoptosis (Figure 2E). These findings confirmed at cellular level the increased cell death observed by both AO or aCas3 staining in the CNS and showed that a cell-autonomous defect of glycine signaling induced NSCs to apoptosis before differentiating into neurons. In addition, we showed that disruption of glycinergic activity in non-neuronal cell does not induce apoptosis (Figures 2B,C; Supplementary Figure 1D). Taken together, these findings confirm the specific survival role of glycine signaling for NSCs and rules-out an apoptotic effect caused by off target toxicity of the MO. Moreover, time-lapse imaging showed that disruption of glycine signaling induced an arrest of INM in NSCs at the G2/M mitosis phases (Gotz and Huttner, 2005) followed by cell death. These results confirmed the increased mitotic M-phase pH3 immunostaining showed previously (Cote and Drapeau, 2012).

#### Glycine signaling is required specifically for survival of nestin^±^ NSCs

Because defects of glycine signaling result in NSC apoptosis, we hypothesized that a subpopulation of NSCs may require glycine signaling to survive. We selected nestin and GFAP as major markers of early embryonic NSCs to analyse these subpopulations. Both *in situ* hybridization and transgenic reporter analyses showed a drastic reduction in the number of nestin^+^ NSCs upon disruption of glycine signaling compared with the GFAP^+^ subpopulation, which appeared unaffected. Additionally, down regulation of p53 expression upon disruption of glycine signaling rescuing partially nestin^+^ NSCs. The nestin protein is a member of the intermediate filament protein family. During embryogenesis it has unequivocally been accepted as a marker of NSCs and used to study their proliferation, migration and differentiation. Functionally, nestin is involved in cytoskeleton dynamics such as maintaining cells shape and regulating cell motility (Rutka et al., 1999; Shi et al., 2016). However, nothing was known about the link between glycine signaling and nestin function. Immunocytochemical and electrophysiological studies of neurospheres derived from postnatal rat striata provided evidence of the presence of functional strychnine-sensitive GlyR in nestin^+^ cells and suggest a possible developmental role of GlyR (Nguyen et al., 2002; Scain et al., 2010). Moreover, several *in vivo* studies performed in zebrafish and mouse models provide clear evidence involving nestin in survival and self-renewal of NSCs (Chen et al., 2010; Park et al., 2010). However, the mechanism by which nestin protects cell survival is still unclear. Recent studies reported that nestin interacts with cyclin-dependent kinase 5 and controls cell functions including apoptosis and cell differentiation (Cicero and Herrup, 2005; Sahlgren et al., 2006), and nestin deletion significantly inhibits the proliferation of colorectal cancer cells by arresting the cell cycle progression at S mitotic phase of division (Li et al., 2015). In support of this finding with nestin^+^ NSCs, a recent study revealed connection between nestin and p53 into liver cancer cells, where they showed that p53-dependent nestin regulation in hepatocellular carcinomas and cholangiocarcinomas cells and this connection regulates cellular plasticity and tumorigenesis in liver cancer (Tschaharganeh et al., 2016).

In summary, our findings identify a developmental role of excitatory glycine signaling in the CNS. It plays a crucial role in survival of NSCs and maintains normal developmental proliferation. Importantly, unlike the GFAP^+^ NSC subpopulation, the nestin^+^ NSC subpopulation was particularly dependent on this activity. We conclude that excitatory glycine signaling helps the survival and proliferation of the nestin^+^ NSC subpopulation as its deficiency leads to the cell cycle arrest and apoptosis of the nestin^+^ subpopulation with a consequent loss of interneurons.

## Ethics statement

This study was carried out in accordance with the recommendations of the guidelines of the Canadian Council for Animal Care. The protocol was approved by the Committee of Research Center of the University of Montreal Hospital Center (CRCHUM).

## Author contributions

AB conducted the experiments and generated the results, PD directed AB's work, AB and PD wrote the manuscript.

### Conflict of interest statement

The authors declare that the research was conducted in the absence of any commercial or financial relationships that could be construed as a potential conflict of interest.

## Supplementary material

Supplementary Figure 1 Development of transplanted NSCs. **(A)** Bright- field image of a dorsal view of neural tube of embryos at 14 hpf (Top panel-right), and transplanted NSCs expressing GFP in the membrane and nucleolus (Top panel-left). **(B)** Selected frames from 1 to 8 h time-lapse sequence of two nearly adjacent transplanted NSCs, which divided after interleukin nucleus migration process (Red Arrow), and generating a neuron shown by RFP expression under HuC promoters (Bottom panel-left). **(C)** mRNA nuclei labeling does not affect survival of NSCs. Confocal z-series images of transplanted NSCs where Ctrl-MO transplanted cells showed a normal development to NSCs (NSCs with green membranes and red nuclei). However, a considerable of apoptotic bodies were observed in Glr-MO transplanted cells (NSCs with green membranes and nuclei). **(D)** Disruption of glycine signaling and mRNA nuclei labeling does not affect survival of non-neuronal. Bright-field image of a lateral view of tail in the top panel and confocal z-series images of Glr-MO cells (green nuclei) green membranes and nuclei in the bottom panel.
